# Acute pancreatitis and pneumonia due to *Mycoplasma pneumoniae*: a case report

**DOI:** 10.1186/s13104-016-2196-y

**Published:** 2016-08-09

**Authors:** Michael Benzaquen, Dan Lebowitz, Pauline Belenotti, Jean-Marc Durand, Jacques Serratrice

**Affiliations:** 1 Service de Dermatologie, Hôpital Nord, Assistance Publique-Hôpitaux de Marseille, Aix-Marseille Université, 13015 Marseille, France; 2 Service de Médecine interne, Hôpital de La Timone, Assistance Publique-Hôpitaux de Marseille, Aix-Marseille Université, 13005 Marseille, France; 3 Service de Médecine interne générale, Hôpitaux Universitaires de Genève, 1205 Geneva, Switzerland

**Keywords:** Acute pancreatitis, Pneumonia, *Mycoplasma pneumoniae*

## Abstract

**Background:**

*Mycoplasma pneumoniae* is a bacterium responsible for 15 to 40 % of acute community-acquired pneumonia in children and 20 % of adult cases. Several extrapulmonary manifestations have been reported. We report a rare case of an adult patient suffering from pneumonia associated with an acute pancreatitis in the setting of *Mycoplasma pneumoniae* infection.

**Case presentation:**

A 28-year-old Caucasian woman was referred for anorexia lasting for 1 week. Her past medical history was notable for congenital hydrocephalus with consecutive ventriculo-peritoneal shunt, epilepsia and paraparesis. The patient rapidly deteriorated, presenting with dyspnea, tachypnea, productive cough, abdominal pain, and onset of fever. C-reactive protein was at 270 mg/L, with a rise in serum lipase (670 UI/L, N: 13–60). A computed-tomography scan showed an acute interstitial edematous pancreatitis without necrosis, consistent with grade C on the Balthazar score. Thoracic sections revealed diffuse parenchymal consolidations combined with ground glass opacities. Calcium and triglyceride levels were normal. There was no history of recent trauma, alcoholic intake or drug intoxication. *Mycoplasma pneumoniae* serological assay showed an elevated IgM titer (22 UA/mL), compatible with recent infection, and cold agglutinins were present. A diagnosis of acute pancreatitis and diffuse interstitial pneumonia caused by an infection with *Mycoplasma pneumoniae* was considered. Respiratory and abdominal evolution was quickly favorable after initiation of clarithromycin 500 mg bid.

**Conclusions:**

The relationship between *Mycoplasma pneumoniae* infection and acute pancreatitis has been debated in the literature for many years. This observation, supported by clinical, biological and radiological features, is an additional argument in favor of a non-fortuitous association.

## Background

*Mycoplasma pneumoniae* (MP) is a bacterium responsible for 15–40 % of acute community-acquired pneumonia in children and 20 % of adults cases [1, 2]. Several extrapulmonary manifestations have been reported: neurological (encephalitis, meningitis, acute polyradiculoneuritis), hematological (autoimmune hemolytic anemia, erythroblastopenia), renal (by deposit of circulating immune complexes), cutaneous (maculo-papular rash, erythema nodosum, urticaria, Stevens–Johnson syndrome) and articular.

The relationship between MP infection and acute pancreatitis has been debated in the literature. In 1973, Mardh et al. reported four adult cases of acute pancreatitis following pneumonia due to MP; in three of the patients, the pancreatitis occurred in the 3rd week after the onset of cough, by which time the respiratory tract symptoms had almost disappeared. The diagnosis of MP infection was based on a rising titer of complement-fixation and metabolism-inhibiting antibodies to the microorganism [3]. One year later, the same authors reported six other patients with pancreatitis occurring 1 or 2 weeks after the onset of pneumonia, two of them being subclinical [4].

In response, this association was questioned by Leinikki et al. who retrospectively evaluated 56 patients with acute pancreatitis: in this population, 18 showed a fourfold or greater increase of MP complement-fixing antibodies, while 28 remained seronegative or had constant titers. In ten patients, the results varied from test to test, indicating anticomplementary activity between the antigen and antibody. The presence of a cross-reaction with pancreatic cellular fragments bearing antigenic similarities with MP was discussed by the authors [5]. In 1978, Freeman and Mc Mahon retrospectively studied paired acute and convalescent serum samples from 27 patients with acute pancreatitis: serological evidence of infection with MP was found in nine out of 27 patients (33 %), independently confirming the hypothetic link considered by Leinikki et al. Four of the nine patients were also shown to have gallstones, for which association with pancreatitis has long been recognized. For these four patients, the authors discussed the possible responsibility of MP, an organism with affinity for cholesterol, to potentiate the effect of gallstones in induction of pancreatitis [6].

MP is a unique cell wall deficient, cholesterol requiring, pleomorphic bacterium, that possesses specialized tip organelle to mediate cytoadherence and thus its pathogenicity. Its role in the worsening of cholesterol-induced atherosclerosis has been widely discussed in clinical research [7]. MP is capable of oxidizing the host cell membrane, thus inducing its apoptosis [8–11]. Those mechanisms could be involved in the pathophysiology of acute pancreatitis induced by MP.

Several recent observations, especially in children, reported acute pancreatitis following MP infection, with or without respiratory symptoms, preceding by a few days the appearance of abdominal pains, sometimes leading to a misdiagnosis of perforated appendicitis [1, 2, 10]. The diagnosis of MP infection was asserted by serological data (complement fixation test [2], passive hemagglutination [5]). More recently, Aram Yang et al. reported the case of a 6-year-old girl with an acute necrotizing pancreatitis, revealed by an altered mental status, 2 days after developing cough, sputum and fever [12]. MP infection was diagnosed with a fourfold increase of MP antibody titer between acute and convalescent sera.

MP infection is associated with the emergence of various autoimmune disorders and autoantibody production, especially cold agglutinins [13]. Cold agglutinins were present in all patients described by Mardh et al. in 1973 [3] and can be considered as an additional argument for MP infection.

We report the unusual case of an adult patient suffering from concomitant pneumonia and acute pancreatitis in the setting of infection with MP.

## Case presentation

A 28-year-old Caucasian woman was referred to our Internal Medicine Department for anorexia lasting for 1 week. Her past medical history was notable for congenital hydrocephalus with consecutive ventriculo-peritoneal shunt, epilepsia and paraparesis. At the time of admission, symptoms were associated with a biological inflammatory response (C-reactive protein, 260 mg/L, N: 0–5), elevated serum lipase (277 UI/L, N: 13–60), aminostransferase (ASAT: 87 UI/L; ALAT: 181 UI/L, N: 0–60), gamma-glutamyl transpeptidase (227 UI/L, N: 0–60), alkaline phosphatase (489 UI/L, N: 30–130), and lactate dehydrogenase (376 UI/L, N < 250), along with hyperleukocytosis (12 G/L, N: 4–10 G/L) and thrombopenia (111 G/L, N: 150–400 G/L). Calcium, triglyceride, serum albumin, urea and glycemia levels were normal. Hypoxemia was noted with a PaO_2_ of 58 mmHg (N: 80–100) on ambient air. Modified Glasgow score for pancreatitis was 1/8 for this patient. There was no history of recent trauma, alcohol intake or drug intoxication. An abdomino-pelvic ultrasound, as well as a computed tomography (CT) scan, showed neither signs of biliary or pancreatic disease, nor gallstones, according to our senior radiologist.

**Fig. 1:**
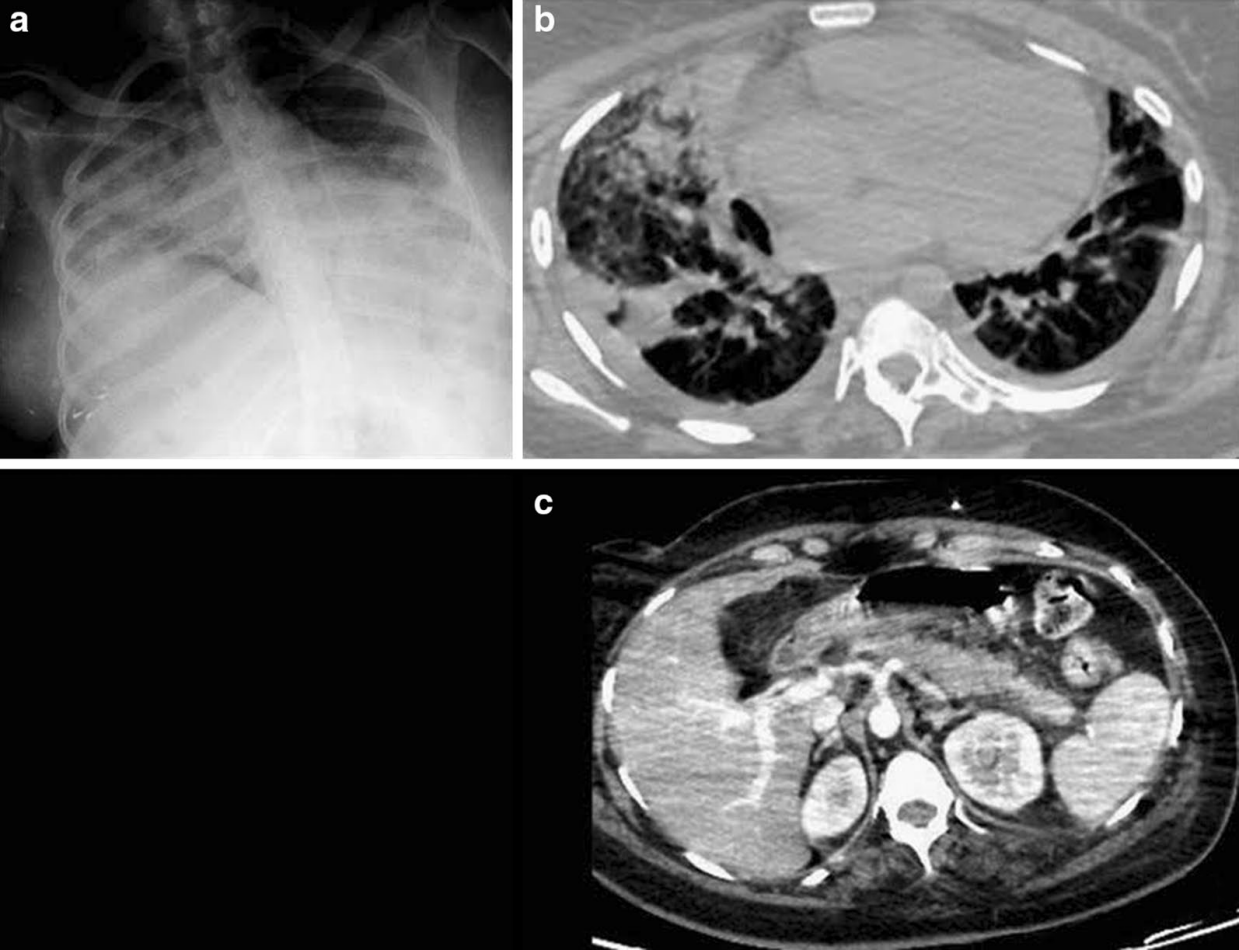
**a** Chest radiograph showing diffuse alveolo-interstitial infiltrates. **b** Chest computed tomography scan showing diffuse parenchymal consolidations combined with ground glass opacities. **c** Abdominal computed tomography scan showing acute interstitial edematous pancreatitis without necrosis, grade C on the Balthazar score

Four days after admission, her condition deteriorated, with dyspnea, tachypnea, productive cough, abdominal pain, and onset of fever. C-reactive protein remained at 270 mg/L, with a rise in serum lipase (670 UI/L, N: 13–60). A chest radiograph showed diffuse alveolo-interstitial infiltrates (Fig. 1a), confirmed by chest CT scan revealing diffuse parenchymal consolidations combined with ground glass opacities (Fig. 1b). An abdominal CT scan showed an acute interstitial edematous pancreatitis without necrosis, consistent with grade C on the Balthazar score (Fig. 1c). Blood cultures, urinary antigens for serogroup 1 *Legionellapneumophila* and *Streptococcus pneumoniae* were negative, as well as serological assays for HIV, hepatitis A, B, C, D, E, *Legionella pneumophila* and *Chlamydia psittaci*. MP serological assay (immunoluminometric assay Liaison, Diasorin^®^) showed an elevated IgM titer (22 UA/mL), compatible with recent infection, and cold agglutinins were present.

A diagnosis of acute pancreatitis associated with diffuse interstitial pneumonia caused by an infection with MP was considered, leading to an adjustment of therapy with initiation of clarithromycin 500 mg bid. Respiratory and abdominal evolution was quickly favorable in a few days, with complete regression of dyspnea, abdominal pain and normalization of serum lipase.

In this observation, anorexia revealed a non-obstructive acute pancreatitis associated with diffuse interstitial pneumonia. Modified Glasgow score was 1/8, consistent with an acute pancreatitis without criteria of severity. We thus believe that the lung disease in our patient is unlikely to be considered as a direct complication of the acute pancreatitis. Moreover, both pancreatitis and pneumonia had a quick and favorable evolution under targeted antibiotic therapy. Despite we could not perform a MP PCR on respiratory secretions, the chronological association between the occurrence of the pancreatitis and the pneumonia, positivity of serological assay, presence of cold agglutinins, lack of other causes for acute pancreatitis and clinical response to clarithromycin are, to our point of view, strongly support considering MP as the etiological agent for both disorders.

## Conclusions

We report a rare case of pneumonia associated with an acute pancreatitis in the setting of MP infection in an adult patient. Although the link between MP and pancreatitis is debated in the literature, this observation, supported by clinical, biological and radiological features, is an additional argument in favor of a non-fortuitous association.

## Abbreviations

MP: *Mycoplasma pneumoniae*
CT: computed tomography
